# Establishment and Characterization of Human Cell Lines Stably Infected With Bovine Leukemia Virus In Vitro Reveals Apoptosis After Latency and Silencing

**DOI:** 10.1155/tbed/6251251

**Published:** 2026-07-29

**Authors:** Samy Metwally, Rania Hamada, Ryosuke Matsuura, Yoko Aida

**Affiliations:** ^1^ Department of Global Agriculture Science, Graduate School of Agricultural and Life Sciences, The University of Tokyo, Tokyo, Japan, u-tokyo.ac.jp; ^2^ Department of Infectious Diseases and Epidemics, Faculty of Veterinary Medicine, Damanhour University, Damanhour City, Egypt, damanhour.edu.eg; ^3^ Department of Pathology and Clinical Pathology, Faculty of Veterinary Medicine, Damanhour University, Damanhour City, Egypt, damanhour.edu.eg

**Keywords:** apoptosis, bovine leukemia virus (BLV), human cell lines, latency, pathogenesis, silent infection

## Abstract

Bovine leukemia virus (BLV) is the etiological agent of B‐cell leukemia/lymphoma in cattle worldwide. Although previous studies have linked BLV to breast cancer in women, its zoonotic potential remains controversial. Moreover, no previous studies have clearly investigated the BLV latency in human cells. In this study, we aimed to establish and characterize human cell lines stably infected with BLV as in vitro models of natural BLV infection. Human epithelial 293T, human cervical cancer HeLa cells (HeLa), and human breast cancer MCF7 cells were infected with cell‐free virus from persistently infected fetal lamb kidney (FLK–BLV) cultures. Single clones of the three infected cell lines were tested every 2 weeks for 18 months. The three infected clones maintained the full‐length BLV provirus. None of the three infected cell lines showed cellular expression of viral gp51 and p24 proteins, cell‐to‐cell infectivity, or detectable virion p24 and RNA throughout the study, indicating BLV silencing in human cells. A significant delay in the doubling time of BLV‐infected 293T and MCF7 cells with cytopathic effects on cellular morphology was observed after BLV latency. The findings revealed that latent BLV‐induced apoptosis in 293T and MCF7 cells but not in HeLa. Additionally, analysis of apoptosis‐related pathways showed that BLV infection did not increase expression of antiapoptotic markers in all infected cell lines. In conclusion, we successfully established three stably infected human cell lines by BLV silencing. Of critical importance to public health, human cells latently infected with BLV undergo apoptosis but not malignant transformation. Further studies to analyze the mechanisms of BLV–human interaction in established models are in progress.

## 1. Introduction

Bovine leukemia virus (BLV), belonging to the *Deltaretrovirus* genus, is the etiological agent of enzootic bovine leukosis (EBL), the most common neoplastic illness in cattle worldwide [1, 2]. BLV is closely related to human T‐cell leukemia virus type 1 (HTLV‐1), which shares similar genomic organization, strategies for gene expression, and pathology; however, BLV affects the B‐cell lineage, while HTLV‐1 affects the T‐cell lineage [2, 3]. In cattle, BLV primarily infects various cell populations; however, mono‐ or poly‐clonal expansion is induced only in BLV‐infected CD5^+^ B cells after a long latency period [2]. B‐cell lymphomas can occur in different organs, resulting in many illnesses that cause animal death [1, 2, 4, 5]. Approximately 70% of cattle infected with BLV show no clinical symptoms, whereas 30% develop persistent lymphocytosis, and 2%–5% of BLV‐infected cattle develop B‐cell lymphoma after long latency periods [2, 6, 7]. Healthy carrier cattle are sources of BLV transmission within the herd through blood‐sucking insects, body fluids, or iatrogenic routes [8]. BLV‐infected cattle with high proviral loads (PVLs) secrete BLV in milk, nasal mucus, and saliva [9–12] and are present in various organs throughout the body of experimentally infected cattle [5]. Additionally, BLV has been detected in beef cattle used for slaughter and in raw beef [13–17]. This suggests that humans continuously exposed to BLV could be at risk of BLV infection [18–23].

Domestic cattle, water buffaloes, and yaks are the natural hosts of BLV [24, 25], with a continuous increase in global BLV prevalence rates [26]. However, other animals such as sheep [27–29], goats [30], pigs [31], rabbits [32], and chickens [33] can be experimentally infected. Some cell lines from various animals and humans were susceptible to BLV infection in vitro [29, 34–36].

Since 1976 [37], several epidemiological studies have reported the presence of BLV in human samples, including peripheral blood, breast tissue, and human cancer cell lines [38]. The earlier BLV studies relied mainly on serological techniques [19, 37, 39, 40], whereas recent studies have adopted more advanced and sensitive techniques designed for detecting the BLV provirus and biomarkers such as viral proteins, thereby enabling a more robust analysis of its relationship with humans [35, 36, 41–46].

Owing to evidence of an association between some viruses and breast cancer tumorigenesis and metastasis [47, 48], most BLV studies have investigated the possible relationship between BLV and breast cancer development in women [36, 42, 43, 46, 49–56]. Several studies have analyzed BLV infection in human blood, blood cell lines, and cancer cells [39, 44, 45, 57–59]. A meta‐analysis of the detection of BLV in human samples reported that 27% of 10,398 samples were BLV‐positive, ranging from 1% to 87% [38]. For example, researchers from the United States have detected the antibodies against BLV p24 in 74% of the tested human sera [40] and reported the detection of the BLV *tax* gene in 44% of human breast cancer tissue samples using in situ polymerase chain reaction (PCR) [60]. High positivity in human samples from various countries, where BLV DNA was detected in breast cancer tissues and blood, with prevalence rates of 30.5% in Brazil [43], 13% in Colombia [23], 22.6% in Argentina [61], 80% in Australia [42], 30% in Iran [59], and 26.8% in Pakistan [46]. These findings suggest that BLV is a human pathogen that is significantly associated with breast cancer in women.

However, researchers from some BLV‐endemic countries cannot detect BLV in human samples. For example, although Japan has a high sero‐prevalence of BLV in cattle and an increase in the occurrence of EBL [16, 62, 63], two studies conducted by our research group showed negative results for BLV detection in Japanese human blood cell lines, human cancer cell lines, human blood samples, and breast cancer tissues [44, 45]. Furthermore, breast cancer tissues, blood samples from healthy patients and patients with leukemia, and lung cancer tissue samples analyzed in several studies in China [64], South Korea [58], Mexico [52], Vietnam [52], and the United States [52, 56, 57, 65] were negative for BLV. These studies hypothesized that BLV is not transmissible to humans and suggested that BLV is unlikely to be a human pathogen.

This controversy has created a significant knowledge gap regarding the zoonotic potential of BLV. To address this, long‐term in vitro studies using a panel of stable BLV‐infected human cell lines are essential to elucidate the viral mechanisms and implications of infection under controlled conditions. Although some human cell lines have shown susceptibility to BLV infection [34–36], certain of the resulting infections were transient [34], with most cells losing the virus within weeks [35], whereas a few cells maintained the virus up to 25 passages [35, 36]. Furthermore, no prior study has demonstrated the stable maintenance of the BLV provirus beyond 3 months or systematically characterized the long‐term effects of infection in human cells. Therefore, this study aimed to establish and characterize stable BLV‐infected human cell lines as an in vitro model to investigate the mechanisms and fate of natural BLV infection in human cells.

## 2. Materials and Methods

### 2.1. Cell Culture and Infection

Human embryonic kidney epithelial 293T cells (293T), human cervical cancer HeLa cells (HeLa), the permanently BLV‐infected fetal lamb kidney FLK‐BLV cells, and human breast cancer MCF7 cells (MCF7) (ATCC HTB‐22) (ATCC, Manassas, VA, USA) and the BLV reporter CC81 transfectants CC81‐GREMG cells [66] were grown in Dulbecco’s modified Eagle’s medium (DMED) (Thermo Fisher Scientific, Waltham, MA, USA) supplemented with 10% heat‐inactivated fetal bovine serum (Sigma–Aldrich, St. Louis, MO, USA), nonessential amino acids (Thermo Fisher Scientific), and penicillin–streptomycin–glutamine (Thermo Fisher Scientific) at 37°C with 5% CO_2_. DMEM was also supplemented with 0.01 mg/mL human insulin (Sigma–Aldrich) for MCF7 culture.

In 24 well‐plates, 10^3^ of human 293T, HeLa, and MCF7 cells were infected with 1 mL filtrated supernatant from FLK–BLV culture containing 10 ng/mL BLV‐capsid p24 after mixing with 1 µg/mL polybrene infection reagent (Sigma–Aldrich) and then incubated for 24 h at 37°C with 5% CO_2_. The supernatant was replaced with a fresh medium, and the cells were incubated for 3 days. Then, the infected cells were continuously passed every 3–4 days. For single cloning, the cells of the fiftieth passage postinfection were harvested and cultured in 96‐well culture plates using dilution, considering one cell per well in a 100 μL culture medium.

### 2.2. DNA Extraction and Quality Testing

Genomic DNA was extracted from human cells using the Wizard Genomic DNA Purification Kit (Promega Corporation, Tokyo, Japan) according to the manufacturer’s instructions. The concentration and quality of the extracted DNA were measured using a NanoDrop One Spectrophotometer (Thermo Fisher Scientific). DNA samples were diluted in nuclease‐free water (NFW) to the desired concentrations for PCR experiments.

For the DNA fragmentation assay, the DNA was extracted from the human cells using the phenol–chloroform extraction reagent (Thermo Fisher Scientific) according to the manufacturer’s instructions.

### 2.3. Nested PCR for Amplification of the Env Gene Fragment of BLV in Human Cells

A partial *env-gp51* gene fragment (598 bp) of BLV was amplified via nested PCR using PrimeSTAR GXL DNA Polymerase (Takara Bio Inc.; Kusatsu, Japan), as described previously [67]. The primer sets Env 4831‐F and Env 5744‐R (listed in Table S1) resulted in the first‐round amplification of the 913‐bp fragment of the *env* gene corresponding to the nucleotide positions 4826–5738 of the full‐BLV genomic sequence recorded in GenBank (Accession Number EF600696) [68]. Then, the gp51‐F 5042 and gp51‐R 5639 primers resulted in the second‐round amplification of the 598‐bp fragment of the BLV *env-gp51* gene corresponding to the nucleotide positions 5036–5634 of the whole BLV genomic sequence.

### 2.4. BLV Provirus Detection by PCR Amplification of a Long Proviral Region

A 100 ng template DNA from each human cell was used to amplify a long fragment (≃8 kb) of the BLV proviral genome using PrimeSTAR GXL DNA Polymerase (Takara), as previously described [45, 69]. The primer set (Table S1) amplifies a 7935‐bp fragment corresponding to the nucleotide positions 741–8676 of the full‐BLV genomic sequence (Accession Number EF600696).

### 2.5. BLV‐CoCoMo‐qPCR‐2 Assay for Evaluation of BLV PVL in Human Cells

The BLV PVL in human cells and the number of BLV RNA copies released from the cells as virions were determined by a BLV‐CoCoMo‐qPCR‐2 assay (Nippon Gene Co., Ltd., Toyama, Japan) using a THUNDERBIRD Probe qPCR Mix (Toyobo, Tokyo, Japan), as described previously [70]. Briefly, either 150 ng of cellular DNA or 500 ng of viral cDNA was used as a template to amplify the BLV long terminal repeat (LTR) gene using the degenerate CoCoMo primer mix targeting the BLV LTR gene and detected using a 6‐carboxyfluorescein (FAM)‐labeled minor groove binder (MGB) probe (Table S1). The amplification was performed on a Light Cycler 480 System II (Roche Diagnostics, Mannheim, Germany). Finally, we expressed the BLV PVL as the number of copies per 150 ng of DNA or 500 ng of cDNA.

### 2.6. Western Blotting

Seventy‐2 h after the culture of BLV‐infected, noninfected negative control (NC) human cells, and FLK‐BLV cells as a positive control (PC), they were harvested and subjected to Western blotting of the cell lysates using anti‐BLV p24 monoclonal antibody (MAb) (BLV‐3; VMRD, Pullman, WA, USA), anti‐BLV gp51 MAb (BLV‐2; VMRD), and anti‐β‐actin MAb, as described previously [71, 72].

### 2.7. BLV p24 Capture ELISA for Detection of Virion Release in the Culture Supernatant

One million BLV‐infected and noninfected 293T, HeLa, and MCF7 cells, or FLK‐BLV cells, were seeded in 10 cm culture dishes and incubated for 72 h at 37°C in 5% CO_2_. The supernatants were collected, centrifuged at 340 ×*g* for 10 min, and filtered through a 0.45 μm pore filter (Millipore, Darmstadt, Germany). Then, the filtrate contents of cell‐free virus were quantified using BLV p24 capture ELISA, as described previously [66, 71].

### 2.8. RNA Extraction From the Cell‐Free Particles and Reverse Transcription

A copy of the filtrated supernatants was used to concentrate the cell‐free particles by centrifugation at 194,000 ×*g* for 45 min at 4°C, as previously described [73]. Then, the sediments containing the cell‐free particles were diluted in 140 µL PBS and subjected to RNA extraction using the QIAamp Viral RNA Mini Kit (Qiagen, Hilden, Germany) according to the manufacturer’s instructions.

Next, DNA remnants were removed from the extracted RNA using the TURBO DNA‐free Kit (Thermo Fisher Scientific), and the final RNA concentration and purity were detected using a NanoDrop One spectrophotometer (Thermo Fisher Scientific). Finally, 2 μg of each RNA sample was reverse‐transcribed into cDNA using the High‐Capacity RNA‐to‐cDNA Kit (Applied Biosystems, Foster City, CA, USA) according to the manufacturer’s instructions.

### 2.9. Luminescence Syncytium Induction Assay (LuSIA)

BLV cell‐to‐cell infectivity in the three BLV‐infected human cell lines was investigated using LuSIA, as described previously [67]. The BLV reporter CC81‐GREMG cells (4 × 10^5^) were co‐cultured with equal numbers of either BLV‐infected and noninfected 293T, HeLa, and MCF7 or FLK‐BLV (2 × 10^5^) cells in 1 mL culture medium in 12 well‐plates and incubated for 72 h at 37°C in 5% CO_2_. The cells were washed with PBS, fixed, and stained in 3.6% formaldehyde/PBS containing 10 μg/mL Hoechst 33342 (Sigma–Aldrich). The stained cells were observed for the enhanced green fluorescent protein (EGFP) expression and fluorescent syncytia formation using an EVOS FL Auto 2 Cell Imaging System (Thermo Fisher Scientific).

### 2.10. Cell Cycle Analysis

Seventy‐2 h after the culture of 10^6^ of BLV‐infected and noninfected 293T and HeLa, or 96 h after the culture of MCF7 in 10 cm culture dishes, the cells were harvested, and the DNA content in the cells was analyzed using flow cytometry as described previously [72]. Briefly, 10^6^ of the harvested cells were washed with PBS and then fixed in 1% formaldehyde and 70% ethanol. The fixed cells were resuspended in RNase A (Invitrogen; 100 μg/mL) at 37°C for 20 min and stained with propidium iodide (PI; Sigma–Aldrich; 50 μg/mL) for 10 min at room temperature. The fluorescence generated by 10,000 events was analyzed using a BD Accuri C6 Plus with a sampler flow cytometer (Becton–Dickinson, Franklin Lakes, NJ, USA). The acquired data were analyzed using FlowJo v10 software (FlowJo, LLC, Ashland, OR, USA).

### 2.11. DAPI Staining and Confocal Laser‐Scanning Analysis

For the detection of apoptosis in the BLV‐infected 293T and MCF7 cells compared to the NCs, the cells were stained with Hoechst 33342 (Sigma–Aldrich) and analyzed using confocal laser‐scanning microscopy as described previously [73, 74]. Briefly, 10^5^ cells were seeded on a coverslip in 12 well‐plates and incubated for 72 h at 37°C in 5% CO_2_, then the cells were washed with PBS, fixed, and stained in 3.6% formaldehyde/PBS containing 10 μg/mL Hoechst 33342 (Sigma–Aldrich). The samples were mounted with 90% glycerol/PBS, and the stained cells were observed for apoptosis and abnormalities using an FV‐1000 confocal laser‐scanning microscope (Olympus, Tokyo, Japan).

### 2.12. Quantitative Reverse Transcription‐PCR (qRT‐PCR)

The total RNA was extracted from 2 × 10^6^ cells using the NucleoSpin RNA isolation kit (MACHEREY‐NAGEL GmbH & Co., Düren, Germany), according to the manufacturer’s instructions. The total RNA concentration and purity were measured using a NanoDrop One Spectrophotometer (Thermo Fisher Scientific), and then 2 μg of each RNA sample was reverse transcribed into cDNA using the High‐Capacity RNA‐to‐cDNA Kit (Applied Biosystems) according to the manufacturer’s instructions.

The primers for the apoptotic genes, such as *caspase 3*, *caspase 6*, and *BAX*; the antiapoptotic gene *Bcl-2*; and the internal control gene (*GAPDH*) were designed using the primer‐designing tool provided by the National Center for Biotechnology Information and listed in Table S2. RT‐PCR was conducted by a KAPA SYBR FAST qPCR Kit (KAPA BIOSYSTEMS, Wilmington, MA, USA) using an Applied Biosystems 7500 Fast Real‐Time PCR system (Applied Biosystems). The thermal condition was as follows: 95°C for 3 min, followed by 40 cycles of 95°C for 10 s and 60°C for 30 s. Each sample was tested in duplicate, and the data were analyzed using the comparative CT method (∆∆CT) with normalization to GAPDH mRNA expression.

### 2.13. Statistical Analysis

The statistically analyzed data were represented as the mean ± standard deviation (SD) for at least 3 independent experiments. The *p*‐values were calculated using a *t*‐test, and *p*‐values of 0.05 or less were considered statistically significant. The figures that included statistically analyzed values were constructed in GraphPad Prism Version 8 (GraphPad Software, San Diego, California, USA).

## 3. Results

### 3.1. Establishment of Three BLV‐Infected Human Cell Lines and Selection of Single Clones

**Figure 1:**
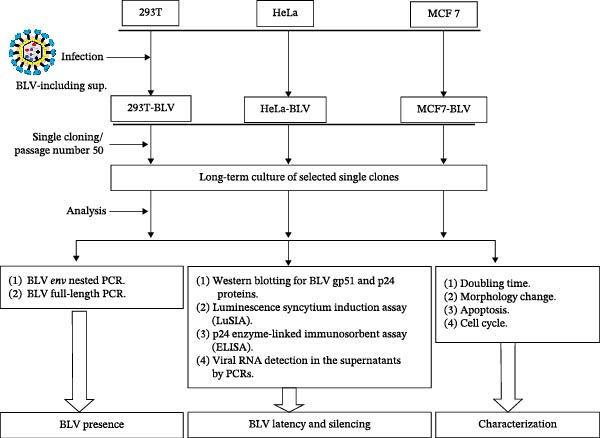
Flowchart of the experimental procedure in this study. Firstly, human 293T, HeLa, and MCF7 cells were infected with filtrated supernatants from FLK–BLV culture containing 10 ng/mL BLV‐capsid p24 after mixing with 1 µg/mL polybrene infection reagent (Sigma–Aldrich). Secondly, single clones from the three infected cells were selected and passed every 3–4 days for 18 months. Thirdly, parallel to the passage, the single clones’ cultures were analyzed every 2 weeks. The BLV provirus was detected by PCRs of the *env* gene and BLV full‐length (≃8 kb) (left panel). Fourthly, western blotting for the expression of viral proteins (gp51 and p24), p24 capture ELISA, and PCRs of viral RNA for virion production and LuSIA for the infectivity of BLV were analyzed for latency and silencing (central panel). Finally, doubling time, morphological change, apoptosis, and cell cycle were analyzed to clarify the mechanism in the infected human cells (right panel).

**Figure 2:**
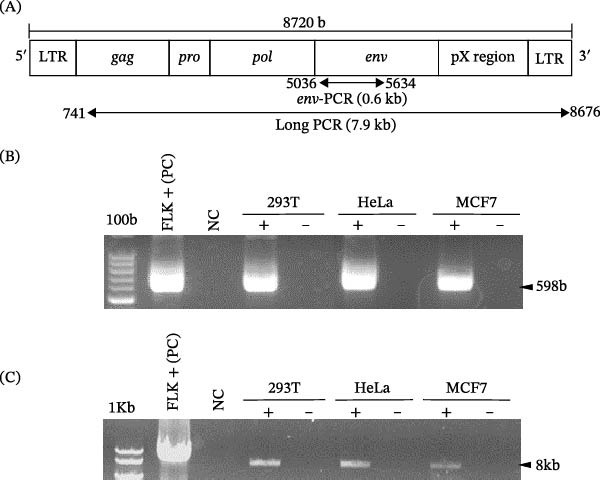
The existence of the BLV provirus in human cells was confirmed by partial *env* gene nested and BLV full‐length PCRs. (A) Schematic presentation of the BLV genomic structure (5’LTR; *gag*, which encodes p15 and p24; *pro*; *pol*; *env*, which encodes gp51 and gp30; and px region ‐3’LTR). (B) Agarose gel (2%) electrophoresis result of PCR products from the second (internal) partial *env* gene‐nested PCR. DNA samples from the three BLV‐infected single clones (+) after latency (18 months) showed a PCR amplicon at 598 bp (indicated by the arrow), while noninfected cells (–) were used as negative controls. PC was DNA obtained from FLK‐BLV cells. NFW was used as a NC for the reaction. The first lane indicates molecular weight (MW) marker (100 bp DNA ladder, [MIXELL Inc., Hiroshima, Japan]). (C) Agarose gel (0.8%) electrophoresis result of PCR products of the BLV full‐length PCR. DNA samples from the three BLV‐infected single clones (+) showed a PCR amplicon at ≃8 kb (indicated by the black arrow), while noninfected cells (–) were used as negative controls. Positive control (PC) was DNA obtained from FLK–BLV cells. NFW was used as a negative control (NC) for the reaction. The first lane indicates the MW marker (1 kb DNA ladder, [MIXELL]).

To demonstrate the effect of BLV on human cells, 293T, HeLa, and MCF7 cells were infected with the supernatant from the FLK‐BLV culture and characterized for the presence of BLV provirus, as described in Figure 1. After BLV infection and passage, the fiftieth passage of the three infected cell lines was used for single clone selection by diluting harvested cells and culturing an individual cell per well in 96‐well plates. Table 1 shows the successful isolation of 10, 14, and 24 single cells from the primary cultures of 293T, HeLa, and MCF7, respectively. The resulting single cells were tested for the presence of the BLV provirus using three PCR assays: partial *env* gene‐nested PCR, BLV full‐length PCR, and CoCoMo‐qPCR‐2. The results revealed that 7 (70%) out of 14 single cells of 293T, 2 (14.3%) out of 14 single cells of HeLa, and 2 (8.3%) out of 24 single cells of MCF7 cells obtained from the primary cultures were positive for the BLV provirus using at least one PCR method. As shown in Figure 1, we successfully selected one single clone from each cell line, such as 293T‐BLV‐3, HeLa‐BLV‐7, and MCF7‐BLV‐8, based on positivity in all three PCR methods (Table 1). The BLV PVLs of these clones were 2730 copies/150 ng DNA for 293T‐BLV‐3, 6180 copies/150 ng DNA for HeLa‐BLV‐7, and 3500 copies /150 ng DNA for MCF7‐BLV‐8. To confirm the presence of BLV in infected cells, genomic DNA from the selected clones was tested every 2 weeks for BLV provirus using partial *env* gene‐nested PCR and BLV full‐length PCR (Table 1 and Figure 2A). After 18 months, nested PCR successfully amplified a 598‐bp fragment of the *env-gp51* gene in all clones (Figure 2B), and full‐length BLV PCR detected a long 7935‐bp fragment in all clones (Figure 2C). Altogether, results from nested PCR of the *env* gene and BLV full‐length PCR confirmed the persistent presence of latent BLV infection in all infected human cell lines.

**Table 1:** Criteria of single clone selection from the three BLV‐infected human cell lines.

Cell line	293T‐BLV	HeLa‐BLV	MCF7‐BLV
Total number of single cells	10	14	24
BLV infection rate (%) ^∗^	7/10 (70)	2/14 (14.3)	2/24 (8.3)
**Characters of selected clone**			
Selected clone’s name	293T‐BLV‐3	HeLa‐BLV‐7	MCF7‐BLV‐8
Partial *env* gene‐nested PCR	+	+	+
BLV full‐length PCR (≃8 kb)	+	+	+
BLV PVL copies/150 ng of DNA	2730	6180	3500

^∗^Cells were tested positive by at least one PCR of partial *env* gene‐nested PCR, BLV full‐length PCR (≃8 kb), or CoCoMo‐qPCR targeting LTRs.

### 3.2. Identification of BLV Silencing in Infected Human Cells

**Figure 3:**
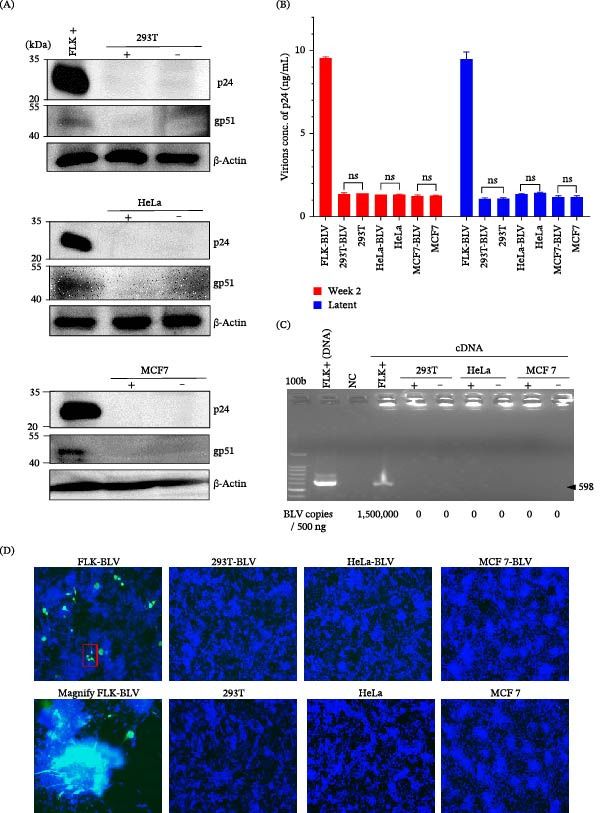
Absence of the expression of cell‐associated viral proteins, the production of viral particles, the release of viral RNA, and cell‐to‐cell infectivity in the three BLV‐infected human cells. (A) Western blot analysis of BLV p24 and gp51 protein expression in the three BLV‐infected human cells. Cell lysates from the three BLV‐infected single clones (+) and noninfected cells (–) after 72 h of culture were prepared and subjected to Western blotting using anti‐p24 (upper panel), anti‐gp51 (middle panel), and anti‐β‐actin (lower panel) antibodies. Cell lysates of FLK‐BLV (FLK+) were used as a positive control. The positions of p24, gp51, and β‐actin are indicated. (B) The production of BLV p24 in the culture supernatants of the three infected human cells with p24 capture ELISA using anti‐BLV p24 MAb. The supernatant from FLK‐BLV culture was used as a positive control. The red columns show the p24 concentration in the supernatants at the second week after infection, while the blue columns show the concentration after BLV latency. Each column and error bar represents the mean ± SD for three independent experiments. The *p*‐values were calculated using a *t*‐test, and ns means a nonsignificant value. (C) No the virion’s RNA release from the three infected human cells. The concentrated cell‐free particles after the ultracentrifugation of 10 mL supernatants of the three BLV‐infected single clones (+) and noninfected cells (–) after 72 h of culture were diluted in 140 µL PBS and subjected to RNA extraction, and DNA was removed. Viral RNA was reverse transcribed, and cDNA was subjected to conventional PCR targeting the amplification of 598 bp of the *env* gene (upper panel) and BLV‐CoCoMo‐qPCR‐2 targeting LTRs of BLV to detect viral RNA (lower panel). The virions from FLK‐BLV culture were used as a positive control, while DNA from FLK‐BLV cells was used as a positive control for the reaction of the conventional PCR. (D) No cell‐to‐cell infectivity in the three BLV‐infected human cell lines using LuSIA. CC81‐GREMG reporter cells, which responded to BLV Tax expression, were co‐cultured with either one of the three BLV‐infected single clones, noninfected negative control cells, or FLK‐BLV cell as a positive control. After 72 h incubation, the cells were fixed and stained with 3.6% formaldehyde/PBS containing 10 μg/mL Hoechst 33342. EGFP‐expressing syncytia were observed using EVOS2 fluorescence microscopy. The merged image is composed of overlaid Hoechst and EGFP images.

To characterize BLV‐infected human cells, western blot analysis of the single clones was performed every 2 weeks to assess the expression of BLV p24 and gp51 proteins (Figure 1). None of the three infected cell lines showed the expression of either viral protein throughout the study period (Figure 3A). In contrast, the PC showed the cellular expression of BLV p24 and gp51 proteins. In addition, β‐actin expression indicated equal loading of cell lysates from each cell line. For further characterization, culture supernatants from the infected cells were investigated for virion release using a BLV p24 capture ELISA (Figure 1). The results showed a complete absence of BLV p24 released from the three BLV‐infected human cell lines throughout the study, from the second week after infection until BLV latency (Figure 3B).

To confirm the absence of virions released from the three infected cell lines, concentrated cell‐free particles in the supernatants were tested for BLV RNA via conventional PCR targeting the *env* gene and using BLV‐CoCoMo‐qPCR‐2 (Figure 1). Conventional PCR targeting a 598‐bp fragment of the *env* gene showed no amplification of BLV RNA at the expected size in any of the three infected cell lines (Figure 3C). Similarly, BLV‐CoCoMo‐qPCR‐2 targeting the LTR detected no copies of BLV RNA per 500 ng cDNA in any of the three infected cell lines, despite the detection of 1.5 million copies of BLV RNA in the PC (Figure 3C).

Using LuSIA, we additionally demonstrated cell‐to‐cell infectivity by assessing syncytia formation induced after co‐culture of the three BLV‐infected human cell lines with CC81‐GREMG reporter cells (Figure 1). Our findings indicate that none of the three infected cell lines showed cell‐to‐cell infectivity or syncytia formation, despite the observed syncytia formation in the PC (Figure 3D).

Western blot analysis, p24 capture ELISA, viral RNA detection, and LuSIA collectively indicated BLV silencing in the three stably infected human cell lines.

### 3.3. BLV Delayed the Cell Doubling Time and Caused Morphological Changes in Human Cells After Latency

**Figure 4:**
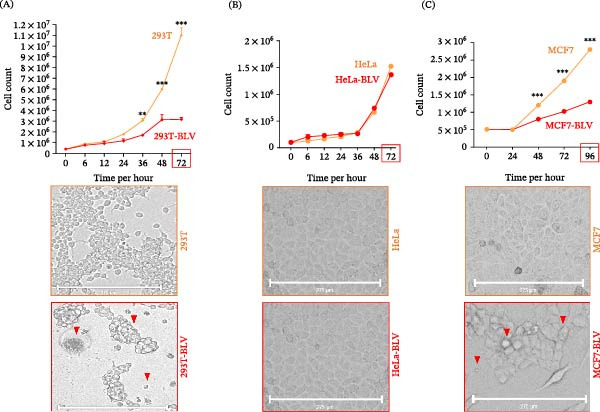
Cell doubling time (upper panels) and growth morphology (lower panels) of the single clones of BLV‐infected 293T (A), HeLa (B), and MCF7 (C) cells after BLV latency. BLV‐infected and noninfected 293T, HeLa, and MCF7 were cultured in 60 mm plates and incubated either for 72 h (A, B) or 96 h (C). Cells were counted manually using trypan blue staining. Each dot and error bar represents the mean ± SD for four independent experiments. The *p*‐values were calculated using a *t*‐test. The asterisk indicates a statistically significant difference ( ^∗∗^ *p* < 0.01 and  ^∗∗∗^ *p* < 0.001). The cell morphology was observed daily using the translight mode of EVOS2 fluorescence microscopy using a 10× lens, and the pictures were selected at the end of the incubation time in each cell as indicated by red squares. The red arrows indicate abnormal cell morphology, colonization, fragmented cells, and loss of demarcation in the infected 293T and MCF7. The scale bars, 275 µm, appear white in the lower part of each picture.

During serial passaging, BLV‐infected 293T cells showed a significant reduction in the cell number (*p* < 0.0001), reaching 3.2 × 10^6^ cells compared with 1.1 × 10^7^ cells in noninfected controls (Figure 4A and Figure 1). A decrease in the cell number was detected after 36 h of culture and became highly significant after 72 h (Figure 4A; upper panel). Consistently, cytopathic effects and altered cellular morphology were first observed in BLV‐infected 293T clones at 7 months post‐cloning (corresponding to passage 60). For example, cytopathic effects were observed after 72 h in BLV‐infected 293T. These cytopathic effects included abnormal cell growth, overlapping colonies, detachment with loss of structural integrity, and fragmentation of colonies, leaving cell debris (Figure 4A; lower panel). Similarly, higher numbers of MCF7 cells were seeded, and cell counts and growth morphology were observed daily for 4 days. The BLV‐infected MCF7 clones showed a significant decrease (*p* < 0.0001) in cell number (1.3 × 10^6^ cells) compared to noninfected cells (2.8 × 10^6^ cells). A decrease in the cell count was detected 48 h after culture, which continued until 96 h (Figure 4C; upper panel and Figure 1). Consistent with the reduced cell numbers, the morphological examination showed clear cytopathic effects and growth abnormalities in BLV‐infected cells compared with those in noninfected controls. BLV‐infected cultures showed abnormal growth, loss of cell boundaries, overlapping colonies, an increased number of floating cells, and fragmentation, leaving cell debris. However, noninfected cells were healthy and confluent (Figure 4C; lower panel). These cytopathic effects and altered cellular morphology were first observed in the BLV‐infected MCF7 clone after 8 months post‐cloning (corresponding to passage 68). In contrast, BLV‐infected HeLa did not show any significant difference (*p* = 0.868) in either cell count (1.5 × 10^6^ cells) or growth morphology compared to their NC cells (Figure 4B and Figure 1).

The doubling times of all BLV‐infected cells and their NCs were calculated. Doubling time analysis (Table 2 and Figure 1) showed a significant delay in the growth of BLV‐infected 293T (30 h) and MCF7 (71 h) cells compared with noninfected 293T (13 h) and MCF7 (38 h) ively. However, the doubling times of BLV‐infected and noninfected HeLa were similar (19 h).

**Table 2:** Doubling time of infected human cells after BLV latency.

Doubling time	293T		HeLa		MCF7	
	BLV−	BLV+	BLV−	BLV+	BLV−	BLV+
Hours ^∗^	13	30	19	19	38	71

^∗^Doubling time was calculated by online software https://www.omnicalculator.com/biology/cell-doubling-time.

### 3.4. BLV Affected Cell Cycle Progression in Infected Human Cells

**Figure 5:**
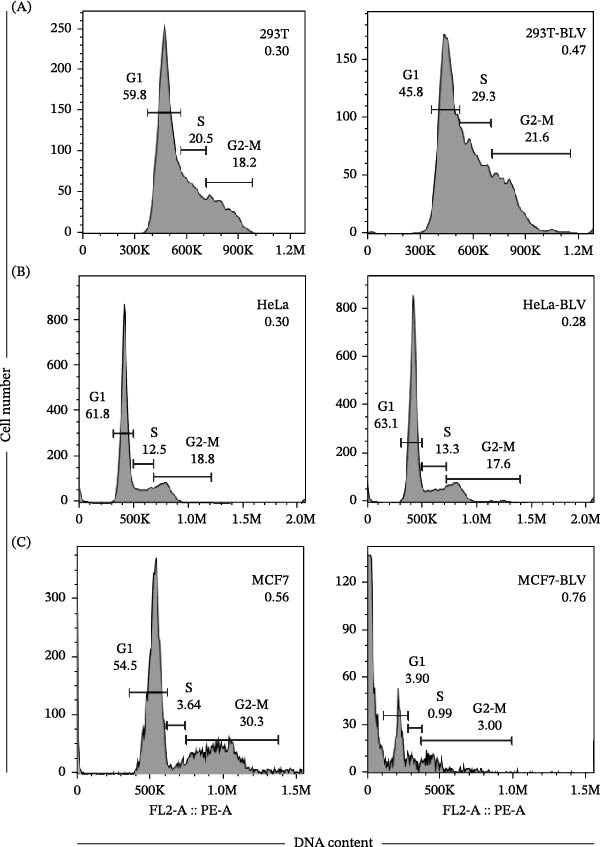
Analysis of the cell cycle and apoptosis using flow cytometry of the BLV‐infected 293T (A), HeLa (B), and MCF7 (C) cells. After 72 h (A, B) or 96 h (C) of culture, cells were harvested, stained using PI, and analyzed using BD Accuri C6 Plus with a sampler flow cytometer (Becton–Dickinson). For each clone, 10,000 events were acquired, and then G1, S, and G2/M phases of the cell cycle were detected by FlowJo v10 software (FlowJo) as indicated on the graphs. Subsequently, the G2/M:G1 ratio was calculated and indicated in the upper part of each graph.

To examine the effects of latent BLV infection in human cells, the DNA content was assessed using flow cytometry after the cells were stained with PI (Figure 1). Data were analyzed, and the G2/M:G1 ratio was calculated for each clone (Figure 5). Analysis of DNA content in 293T cells at 72 h post‐cultivation revealed significant differences between BLV‐infected and noninfected 293T cells. In the BLV‐infected 293T, the G2/M:G1 ratio was 0.47 compared to 0.30 in the noninfected cells. This increase was associated with a decrease in the proportion of cells in G1 (45.8%) in BLV‐infected cells vs. 59.8% in controls, whereas the proportion in G2/M remained unchanged (Figure 5A). In contrast, BLV‐infected HeLa did not show a significant change in DNA content compared to the noninfected cells, with G2/M:G1 ratios of 0.28 and 0.30, respectively. Moreover, the proportion of BLV‐infected HeLa in G1 (63.1%) and G2/M (17.6%) was similar to those in G1 (61.8%) and G2/M (18.8%) of noninfected cells (Figure 5B). Analysis of DNA content in MCF7 cells at 96 h post‐cultivation revealed a significant difference between the BLV‐infected and noninfected cells, where the majority of BLV‐infected cells underwent apoptosis. The G2/M:G1 ratio was increased, reaching 0.76 in BLV‐infected cells compared with 0.56 in noninfected MCF7 cells (Figure 5C).

Overall, cell cycle analysis showed an increased G2/M:G1 ratio in BLV‐infected 293T and MCF7 clones, accompanied by a marked decrease in the proportion of cells in G1 compared with noninfected cells. These findings suggest that BLV induces apoptosis in both cell lines after latency, even without inducing G2 arrest. However, a similar phenomenon was not observed in the BLV‐infected HeLa.

### 3.5. BLV Induces Apoptosis in Infected Human Cells

**Figure 6:**
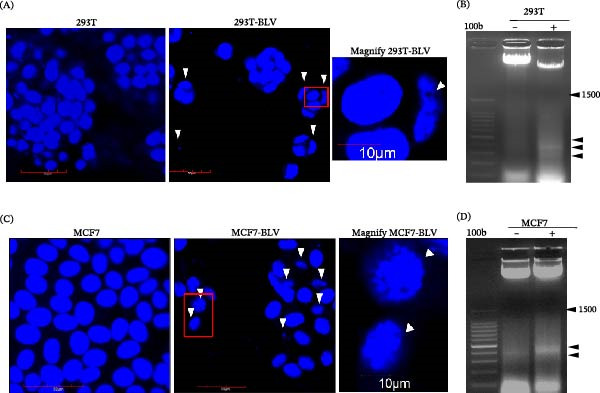
Detection of apoptosis using confocal laser‐scanning analysis and DNA fragmentation assay in the BLV‐infected 293T cells (A, B) and MCF7 cells (C, D). (A, C) Cells were seeded on a coverslip in 12 well‐plates and incubated for 72 h. After staining with 3.6% formaldehyde/PBS containing 10 μg/mL Hoechst 33342, the stained cells were observed for apoptosis and abnormalities using an FV‐1000 confocal laser‐scanning microscope. The white arrows indicate apoptotic bodies with abnormal shapes of nuclei, and the red square areas were magnified (×5) to clearly show the apoptotic cells. The scale bars, 50 µm or 10 µm, appear in the lower left of each picture. (B, D) After 72 h of culture, DNA was extracted from the BLV‐infected single clones (+) and noninfected cells (–) using the phenol‐chloroform method. The stained DNA (20 µg) was run in a 1.5% agarose gel at 100 volts for 40 min and then visualized by UV light. DNA fragmentation was shown in the BLV‐infected cells (+) as indicated by black arrows on the right side of each picture. The first lane indicates MW marker (100 bp DNA ladder).

To confirm that BLV‐induced apoptosis in BLV‐infected 293T and MCF7 clones after latency and after 72 h of cultivation, both cell lines were further analyzed using confocal laser‐scanning microscopy and a DNA fragmentation assay (Figure 1). The presence of apoptotic cells in BLV‐infected 293T cultures was indicated by abnormal crescentic‐shaped nuclei, nuclear condensation, fragmentation, and chromatin clumping after Hoechst 33342 staining (Figure 6A). Consistently, gel electrophoresis of extracted DNA from the BLV‐infected 293T cells showed DNA fragmentation, with multiple fragments smaller than 500 bp, and reduced intact genomic DNA compared with noninfected cells (Figure 6B). In BLV‐infected MCF7 cells, apoptotic cells were identified by the presence of small shrunken nuclei, nuclear condensation, fragmentation, and chromatin clumping observed using confocal laser‐scanning microscopy (Figure 6C). Similarly, gel electrophoresis of extracted DNA from BLV‐infected MCF7 cells showed DNA fragmentation, with clear fragments of less than 500 bp (Figure 6D).

### 3.6. Mechanisms of BLV‐Induced Apoptosis in Human Cells

**Figure 7:**
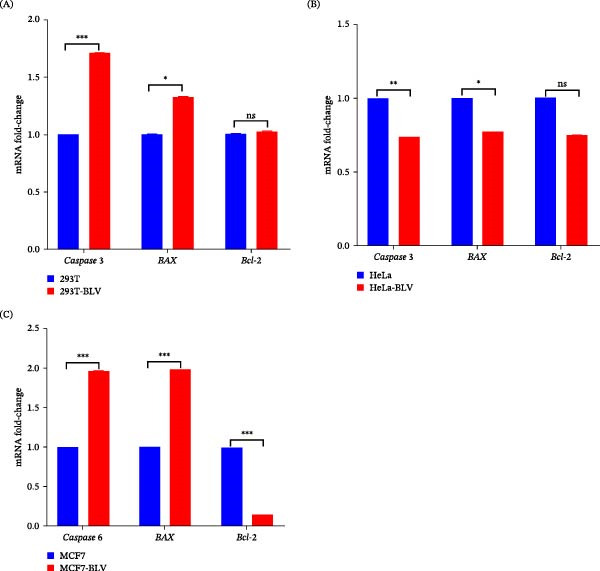
Quantitative analysis of the mRNA expression levels of apoptotic (*caspase 3*, *caspase 6*, and *BAX*) and antiapoptotic (*Bcl-2*) genes in the BLV‐infected 293T (A), HeLa (B), and MCF7 (C) cells. After 72 h of culture, the total RNA was extracted from the cells and reverse transcribed. Each gene expression was measured in the cDNA samples using qRT‐PCR, and then the fold‐change was calculated using the ΔΔCT method with normalization to GAPDH expression as an internal control. Each column and error bar represents the mean ± SD for three independent experiments. The *p*‐values were calculated using a *t*‐test and the asterisk indicates a statistically significant difference ( ^∗^ *p* < 0.05,  ^∗∗^ *p* < 0.01, and  ^∗∗∗^ *p* < 0.001), while ns means a nonsignificant value.

Analysis of apoptosis‐related gene expression was performed to confirm the findings shown in Figure 6 and to provide insights into the mechanisms of apoptosis in human cells. Therefore, the mRNA expression levels of proapoptotic genes (*caspase 3*, *caspase 6*, and *BAX*) and antiapoptotic genes (*Bcl-2*) were quantified using qRT‐PCR. The BLV‐infected 293T cells showed a significant increase in the mRNA levels of apoptotic genes compared with those of noninfected 293T cells. The fold‐change of *caspase 3* was 1.72 (*p* = 0.0015), whereas that of *BAX* was 1.33 (*p* = 0.025). However, the difference in antiapoptotic *Bcl-2* gene mRNA level was not significant (fold change 1.03, *p* = 0.428) (Figure 7A). Similarly, the mRNA levels of the same three genes were quantified in BLV‐infected HeLa. The results indicated a slight decrease in the mRNA expression of all tested genes compared with that in noninfected HeLa, with fold‐changes of 0.74 (*p* = 0.007), 0.77 (*p* = 0.04), and 0.75 (*p* = 0.054) for *caspase 3*, *BAX*, and *Bcl-2*, respectively (Figure 7B). In BLV‐infected MCF7 cells, mRNA expression of *caspase 6*, *BAX*, and *Bcl-2* genes was quantified. The BLV‐infected MCF7 clone showed a significant increase in the mRNA levels of apoptotic genes compared to those of noninfected MCF7 cells. The *caspase 6* and *BAX* fold‐changes were 1.97 (*p* = 0.003) and 1.99 (*p* = 0.002), respectively. In contrast, mRNA expression of *Bcl-2* was significantly decreased in BLV‐infected cells compared with noninfected MCF7 cells (fold change 0.14, *p* = 0.0015) (Figure 7C).

### 3.7. Comparison of the Effects of BLV in Three Stably Infected Human Cell Lines

Similar to BLV‐infected 293T and MCF7 cells, the HeLa cell line retained the BLV provirus throughout the experimental period. BLV silencing was observed in all three cell lines. However, unlike the other two cell lines, HeLa exhibited no alterations in doubling time or cellular morphology, suggesting resistance to BLV‐induced apoptosis (Table 3). Based on the qRT‐PCR findings, differences in apoptosis‐related gene expression suggested distinct mechanisms of BLV‐induced apoptosis among the cell lines. In 293T cells, apoptosis was associated with increased transcription of apoptotic genes without a significant change in antiapoptotic gene expression. In MCF7 cells, apoptosis was associated with increased transcription of apoptotic genes and a highly decreased transcription of antiapoptotic genes. In contrast, BLV did not induce apoptosis in HeLa, consistent with the relatively balanced expression of apoptotic and antiapoptotic genes.

**Table 3:** Summary of the results of BLV‐infected human cells.

Single clone	BLV existence	Viral protein expression	Virion release	Cell‐to‐cell infectivity	Doubling time	Cell morphology	Apoptosis			
								*Caspases*	*BAX*	*Bcl-2*
293T‐BLV	+	–	–	–	Delay	Abnormal	+	⇧	⇧	⬄
HeLa‐BLV	+	–	–	–	No change	Normal	–	⇩	⇩	⬄
MCF7‐BLV	+	–	–	–	Delay	Abnormal	+	⇧	⇧	⇩

*Note:* The arrow (⇧) means a significant increase, the arrow (⇩) means a significant decrease, while (⬄) means a nonsignificant.

## 4. Discussion

The findings supported four major conclusions. First, we generated three stable human BLV‐infected cell lines using a cell‐free infection model. These cell lines maintained the full‐length BLV provirus throughout the experimental period, representing the longest duration reported to date and consistent with BLV latency. Second, BLV latency was associated with BLV silencing in all infected human cells, as indicated by the absence of viral protein expression, cell‐to‐cell infectivity, and virion‐associated p24 and RNA. Third, this study is the first to report the induction of apoptosis in two latent BLV‐infected human cell lines. Fourth, analysis of apoptosis‐related pathways showed that BLV infection did not increase the expression of antiapoptotic markers, which may indicate its inability to induce leukemogenic transformation in the three infected cell lines.

Previous studies reporting the susceptibility of human cells to BLV infection in vitro [29, 34–36, 74–76], the presence of BLV in human tissues and blood [39, 57–60], and evidence suggesting a relationship between BLV and breast cancer in women [36, 42, 43, 46, 49–56] encouraged us to establish stable BLV infection models in human cells. This study used three human cell lines: embryonic kidney epithelial 293T cells as a representative epithelial model, cervical cancer HeLa as a cancer model, and breast cancer MCF7 cells as a human breast cancer model. The susceptibility of these or similar cell types to BLV infection has been reported previously [29, 34, 35, 74, 76]. Cellular susceptibility to BLV infection is mediated by expression of receptors for BLV, including cationic amino acid transporter 1/solute carrier family 7 member 1 (SLC7A1/CAT‐1) [74] and AP3D1 [77] proteins. Overexpression of the BLV receptor SLC7A1/CAT‐1 enhanced cellular susceptibility to BLV infection [74, 78].

We used a BLV cell‐free infection model [34, 75, 76, 79, 80] to infect human cells with BLV virions. Despite the lower infection efficiency of this model, it offers several advantages over cell‐to‐cell infection models used in other studies [35, 36]. This model protects target cells from contamination with the remnants of the BLV‐infected cells and avoids the influence of cell‐to‐cell transmission on disease progression, as previously reported for HTLV‐1 [81, 82]. In addition, it allows for infected cell characterization during the early BLV infection stages, avoiding the use of peripheral blood mononuclear cells that would require several passages for complete cleaning from the culture [36]. Similar to other retroviruses, BLV entry into cells is initiated by the attachment of the viral envelope glycoprotein to the host cellular receptors. Viral RNA is released into the cytoplasm and reverse‐transcribed by the viral‐encoded enzyme reverse transcriptase to produce viral DNA, which is then transported to the cell nucleus, where it is integrated into the cellular genome as a provirus through the viral‐encoded integrase enzyme to generate a stable infection [2, 74, 83]. A wide variation in the BLV infection rate among the three infected cell lines has been described, despite the cells being exposed to the same infective dose of BLV virions. The observed difference is likely due to the varying levels of the BLV receptor SLC7A1/CAT‐1 [74, 78]. An additional factor may be inefficient transport of the reverse‐transcribed viral DNA from the cytoplasm to the nucleus [76]; DNA may be rapidly degraded in the cytoplasm of infected cells [84]. This supports the expectation that MCF7 cells harbor fewer copies of BLV than other cell types [35]. Cell division, cell death, and clonal expansion of infected cells may contribute to these differences [85]. These results demonstrate that the cell‐free virus can complete the primary steps of the BLV life cycle, including viral attachment, entry, and RNA reverse transcription, in three types of infected human cells with different degrees of success.

Stable BLV infection of the three cell lines was confirmed by detecting the BLV provirus genome in DNA samples extracted from single‐cell clones throughout the study. Several regions of the BLV genome were targeted for the investigation and follow‐up of the existing BLV provirus. Nested PCR targeted a conserved region of the genome, the *env-gp51* gene, which is used in BLV genotyping [69], whereas the highly sensitive BLV‐CoCoMo‐qPCR‐2 targeted the LTR region [70]. Additionally, a BLV long fragment (≃8 kb) was successfully detected using the conventional PCR [45, 69]. In our future studies, we aim to sequence the full‐length PCR products from the amplified long fragments to identify possible mutations occurring in the BLV genome as mutations in both the BLV LTR and *tax* genes can affect promoter activity and consequently influence PVL, viral fitness, and transmissibility of BLV in cattle [86]. This is the longest study to follow BLV infection and persistence in human cells, suggesting that these three established cell lines are suitable models for studying BLV latency in humans. Previous studies [35, 36] investigated BLV‐infected human cells for shorter periods, which may be insufficient to reveal the biological mechanisms and pathologies of BLV in human cells. Furthermore, Olaya‐Galan et al. [35] reported that DLDI, 293T, Raji, MCF‐102 A, and HS‐27 human cells were unable to maintain BLV postinfection and that BLV DNA decreased with time and became undetectable, although the mechanism remained unclear. In contrast, our three infected cell lines, including 293T cells, maintained the BLV provirus throughout the study. This apparent discrepancy related to BLV existence in 293T cells could be attributed to our distinct infection protocol, applying a high cell‐free virus concentration, achieving a 70% initial infection rate, combined with subsequent single‐cell cloning to permanently maintain 100% proviral integration. Overall, detection of BLV confirms the persistence and stability of BLV in infected human cells and the successful establishment of these human cell line models. Moreover, these findings further support the hypothesis that BLV may act as a zoonotic viral agent.

BLV silencing was observed in all BLV‐infected human cells throughout the study. The absence of viral protein expression, cell‐to‐cell infectivity, p24, and virion RNA indicated BLV silencing. BLV silencing and the subsequent maintenance of viral latency play essential roles in establishing persistent infection in vivo [86]. Consistent with previous findings, human diploid embryonic lung Wl‐38 cell cultures produced low amounts of virus only during the first two passages after cell‐free infection despite remaining infected [75]. Similarly, some acutely infected cells silence viral transcription, becoming latently infected and forming HIV reservoirs in vivo. Latently infected cells persist despite antiretroviral therapy and are a major barrier to the HIV cure [87]. In contrast to our findings, human mammary epithelial cells (MCF‐10A), infected using the cell‐to‐cell model, expressed p24 protein and produced infectious BLV particles [36]. However, BLV transfection [34] and cell‐to‐cell infection expose target cells to high viral loads and potential cross‐contamination from infected cells [80]. BLV silencing is thought to play an important role in allowing the virus to evade the host’s immune defenses [88]. In BLV and HTLV, 5ʹ ‐LTRs play a major role in controlling viral gene expression, with the regulation of transcription initiation being a major mechanism of silencing [86]. Several mechanisms of BLV silencing, including viral and host factors, have been reported, such as epigenetic modifications of the provirus, LTR transactivator proteins, hormonal signaling, and the interaction of antisense transcripts with viral microRNAs [86, 88–94]. In contrast, retroviral reactivation is a major target for treating infected patients as virus‐producing cells are selectively depleted by antiretroviral therapy [95]. Several agents capable of inducing HIV‐1 latency reversal and therapy have been identified [95]. Regarding BLV latency reactivation, our earlier study showed the reactivation of BLV in the blood of infected cows [88]. Moreover, exposure to 17‐β estradiol increased p24 expression and PVL in infected MCF‐10A human cells [36]. Understanding the mechanisms driving BLV latency and latency reversal in human cell models should be the focus of future studies.

We observed apoptosis in the BLV‐infected 293T and MCF7 cells after a long latency period. To the best of our knowledge, this is the first report on apoptosis in human BLV‐infected cells. Both cell lines exhibited morphological changes and cytopathic effects induced by BLV after 7–8 months. Consistent with previous studies, BLV‐infected human cells showed no morphological changes for up to 3 months [35, 36]. In contrast, none of the three infected human cell lines in our study showed BLV‐induced malignant transformation, as expected in another study [36]. This finding supports the importance of long‐term investigation for characterizing BLV in human cells. Experimentally, BLV induces cytopathic effects and apoptosis in a bovine mammary epithelial cell line (MAC‐T) at the third passage postinfection [96]. In naturally infected cattle, BLV causes marked changes in lymphocyte populations accompanied by alterations in proliferation and apoptosis [97]. Naturally infected cattle with low PVLs present an increased apoptosis rate in their blood and somatic milk cells compared to that of cattle with high PVLs [98, 99].

Apoptosis is essential for maintaining cellular homeostasis, and the failure of cells to undergo apoptosis contributes to cancer development [100]. BLV infection disturbs the equilibrium between proliferation and apoptosis [7]. Several mechanisms underlying BLV‐induced apoptosis have been reported. For example, the expression of the BLV Tax protein can induce apoptosis by activating the molecules associated with apoptosis [29, 101]. When 293T cells were transiently transfected with either wild‐type or mutant BLV, apoptosis was induced [29]. MicroRNA expression in B cells derived from infected Japanese Black cattle revealed that BLV‐derived miRNAs accounted for 38% of all miRNAs in B cells [102]. Additionally, the BLV‐derived long noncoding RNA AS1‐S, a major transcript expressed in latently infected cells, affects host mRNAs [94]. miRNAs can induce apoptosis in PC12 cells BY reducing *Bcl-2* [103] and in other human cancer cells through the mitochondrial caspase cascade and *Bcl-2* pathways [104].

In our study, the cell cycle profiles of 293T‐BLV and MCF7‐BLV appear qualitatively different, with 293T‐BLV cells primarily exhibiting cell redistribution from G1 to the S and G2/M phases, without a clear increase in the sub‐G1 fraction, whereas MCF7‐BLV cells display a prominent sub‐G1 population, a pattern potentially reflecting distinct underlying BLV infection‐affected biological processes. Moreover, the inherent characteristics of these cell lines (293T epithelial cells vs. MCF7 breast cancer cells) likely play a major role in how they respond to BLV latency. However, further comparative analysis of the biological pathways affected in both cell lines would be required. In addition, BLV‐infected 293T and MCF7 cells showed significant variations in the expression of caspases and *Bcl-2* family genes, which regulate apoptotic signaling [105]. The increased mRNA expression of apoptotic genes and decreased expression of antiapoptotic genes may indicate the mechanism of BLV‐induced apoptosis in both cell lines. A similar mechanism involving the caspase cascade pathway of apoptosis without decreasing antiapoptotic *Bcl-2* has been reported in 293T cells transiently transfected with BLV [29]. In MCF7 cells, apoptosis was associated with activation of the caspase cascade pathway and increased *BAX* expression accompanied by decreased *Bcl-2* expression. This represents a typical apoptotic pathway in MCF7, as previously reported [100]. Because of the lack of *caspase 3* in MCF7 cells, the mRNA of *caspase 6* was quantified to demonstrate the caspase cascade pathway of apoptosis [100]. In naturally infected cattle, BLV activates the *Bcl-2* pathway in blood and somatic milk cells to induce apoptosis [99]. In contrast to 293T and MCF7, cytopathic effects and apoptosis were not observed in BLV‐infected HeLa, and these cells appear to maintain apoptotic homeostasis with a minimal effect on the apoptosis pathways. HeLa were developed from a particularly aggressive cervical cancer cell strain and are known to exhibit substantial resistance to apoptosis [106], which may explain this observation. Indeed, previous microarray‐based gene expression analysis revealed that the BLV transcriptional activator Tax protein exerts expression as a stress response beyond host cell transcription, signaling, and immune response in HeLa [101]. In particular, BLV Tax has a possibility to regulate heat‐shock proteins (HSPs) such as DNAJB1, HSPA1A, and HSPA6; the JNK signaling and p38 MAPK signaling pathways, thereby inhibiting apoptosis in HeLa [101]. Moreover, it might be explained by recent evidence that BLV engages unknown different mechanisms in cancer patients, distinct from those employed in infected cattle [107]. Apoptosis in latently infected human cells was confirmed using multiple approaches, including monitoring cytopathic effects by light and confocal laser‐scanning microscopy, doubling time calculation, cell cycle analysis, DNA fragmentation assays, and quantitative detection of mRNA of apoptotic and antiapoptotic genes. Finally, although BLV has been implicated in the development of human breast cancers in previous studies [36, 42, 43, 46, 54, 55], our results showed a contrasting pattern, with BLV inducing apoptosis in epithelial and breast cancer cell lines after latency. Even in HeLa, BLV did not increase the levels of antiapoptotic markers.

Despite the importance of our findings, a primary limitation of this study is that our results are derived exclusively from in vitro human cell models, making our hypothesis regarding BLV’s potential zoonotic role that must be validated by clinical data. Furthermore, our analysis of apoptotic pathways was restricted to mRNA expression profiles, leaving potential post‐transcriptional regulatory mechanisms incompletely understood due to the lack of the corresponding protein expression analysis.

In conclusion, we established three human cell lines stably infected with the BLV. These cell lines represent a novel model for studying BLV infection in humans and provide insights into the implications, biological mechanisms, and pathology of BLV in the human body. Our results are consistent with the possibility that BLV acts as a zoonotic agent; however, we provide new evidence that BLV induces apoptosis after a long latency in human cells without causing malignant transformation. Further studies on the mechanisms of BLV silencing, the impact of BLV reactivation with regard to the possibility that the virus could be derepressed following infection of human cells, and host factors targeting BLV in human cell models are currently in progress.

## Author Contributions

Conceptualization, methodology, validation, writing – original draft preparation, writing – review and editing, funding acquisition: Yoko Aida and Samy Metwally. Formal analysis: Samy Metwally. Investigation: Samy Metwally, Rania Hamada, and Ryosuke Matsuura. Resources, supervision: Yoko Aida.

## Funding

This study was supported by a Grant‐in‐Aid for JSPS Fellow (Grant 24KF0105) from the Japan Society for the Promotion of Science (JSPS) and by a grant from the Livestock Promotional Subsidy from the Japan Racing Association (JRA).

## Disclosure

All authors have read and agreed to the published version of the manuscript.

## Ethics Statement

This study was approved and reviewed by the Research Ethics Committee of the University of Tokyo (Approval Number L21–021H03).

## Conflicts of Interest

The authors declare no conflicts of interest.

## Supporting Information

Additional supporting information can be found online in the Supporting Information section.

## Supporting information


**Supporting Information** The following supporting information includes Table S1: The primers used for the detection of BLV in the BLV‐infected human cell lines. Table S2: The primers used for quantitative real‐time PCR for the detection of the gene expression in the BLV‐infected cells.

## Data Availability

The original contributions presented in the study are included in the article; further inquiries can be directed to the corresponding author.
