# Supplementary material for: Establishment and Characterization of Human Cell Lines Stably Infected With Bovine Leukemia Virus In Vitro Reveals Apoptosis After Latency and Silencing
Source: Transbound Emerg Dis. 2026 Jul 29;2026:6251251. doi: 10.1155/tbed/6251251 (PMC13417729; doi:10.1155/tbed/6251251)
Supplement: Supplementary file 1 — Supporting Information The following supporting information includes Table S1: The primers used for the detection of BLV in the BLV‐infected human cell lines. Table S2: The primers used for quantitative real‐time PCR for the detection of the gene expression in the BLV‐infected cells. [file TBED-2026-6251251-s001.docx]

**Table S1**. The primers used for the detection of BLV and its integration sites in the BLV-infected human cell lines

| **Target** | **Experiment** | **Primer name** | **Position** | **Sequence (5’ – 3’)** | **Reference** |
| --- | --- | --- | --- | --- | --- |
| ***env* gene** | First PCR (913 nt) | Env 4831-F | 4,831 – 4,849 | ATGCCYAAAGAACGACGG |  |
|  |  | Env 5744-R | 5742 – 5744 | CGACGGGACTAGGTCTGACCC | [67] |
|  | 2nd PCR (598 nt) | gp51- F 5042 | 5,036 – 5,058 | TCTGTGCCAAGTCTCCCAGATA |  |
|  |  | gp51- R 5639 | 5,613 – 5,634 | AACAACAACCTCTGGGAAGGGT |  |
|  |  |  |  |  |  |
| **BLV-full length** | Long PCR (7935 nt) | 741-F | 741-765 | CCGATTTAAAGAATTACATCCATTG | [69] |
|  |  | LTR-R | 8,654-8,676 | GCGAGAAACAGAAAGTAAGACAGG |  |
|  |  |  |  |  |  |
| **LTRs** | CoCoMo-qPCR-2 | Primer-Fw | Both were mixed in 1:10 | AATCCMNMYCYKDAGCTGCTGAYYTCACCT  ATCCACACCCTGAGCTGCTGCACCTCACCT | [70] |
|  |  | Primer-Rv |  | TTGCCTTACCTGMCSSCTKSCGGATAGCCGA |  |
|  |  | Probe |  | FAM-CTCAGCTCTCGGTCC-NFQ-MGB |  |

*Binding position refers to bovine leukemia virus cell-line FLK-BLV sub-clone pBLV913 complete genome (Accession number: EF600696).

**Table S2**. The primers used for quantitative real-time PCR for the detection of the gene expression in the BLV-infected cells

| **Experiment** | **Gene** | **Primer name** | **Sequence (5’ – 3’)** | **Fragment length (bp)** |
| --- | --- | --- | --- | --- |
| **Internal control** | *GAPDH* | GAPDH-F | TTGGCTACAGCAACAGGGTG |  |
|  |  | GAPDH-R | GGGGAGATTCAGTGTGGTGG | 161 |
|  |  |  |  |  |
| **Apoptosis** | *Caspase 3* | Caspase 3-F | ATGGAAGCGAATCAATGGA |  |
|  |  | Caspase 3-R | TGTACCAGACCGAGATGTC | 137 |
|  | *Caspase 6* | Caspase 6-F | CGATGTGCCAGTCATTCCTT |  |
|  |  | Caspase 6-R | CTCTAAGGAGGAGCCATAT | 229 |
|  | *BAX* | BAX-F | TGGCAGCTGACATGTTTTCTGAC |  |
|  |  | BAX-R | TCACCCAACCACCCTGGTCTT | 195 |
|  | *Bcl-2* | Bcl-2-F | TCGCCCTGTGGATGACTGA |  |
|  |  | Bcl-2-R | CAGAGACAGCCAGGAGAAATCA | 134 |
